# The clinical manifestations, molecular mechanisms and treatment of craniosynostosis

**DOI:** 10.1242/dmm.049390

**Published:** 2022-04-22

**Authors:** Eloise Stanton, Mark Urata, Jian-Fu Chen, Yang Chai

**Affiliations:** 1 Center for Craniofacial Molecular Biology, University of Southern California, Los Angeles, CA 90033, USA; 2 Keck School of Medicine, University of Southern California, Los Angeles, CA 90033, USA; 3 Division of Plastic and Maxillofacial Surgery, Children's Hospital Los Angeles, Los Angeles, CA 90033, USA

**Keywords:** Craniosynostosis, Human genetics, Environmental factors, Animal models, Mesenchymal stem cells, Tissue regeneration

## Abstract

Craniosynostosis is a major congenital craniofacial disorder characterized by the premature fusion of cranial suture(s). Patients with severe craniosynostosis often have impairments in hearing, vision, intracranial pressure and/or neurocognitive functions. Craniosynostosis can result from mutations, chromosomal abnormalities or adverse environmental effects, and can occur in isolation or in association with numerous syndromes. To date, surgical correction remains the primary treatment for craniosynostosis, but it is associated with complications and with the potential for re-synostosis. There is, therefore, a strong unmet need for new therapies. Here, we provide a comprehensive review of our current understanding of craniosynostosis, including typical craniosynostosis types, their clinical manifestations, cranial suture development, and genetic and environmental causes. Based on studies from animal models, we present a framework for understanding the pathogenesis of craniosynostosis, with an emphasis on the loss of postnatal suture mesenchymal stem cells as an emerging disease-driving mechanism. We evaluate emerging treatment options and highlight the potential of mesenchymal stem cell-based suture regeneration as a therapeutic approach for craniosynostosis.

## Abstract (precis)

**Summary:** Craniosynostosis has broad clinical manifestations and a complex etiology. We review studies from humans and animal models and discuss the developmental mechanisms of craniosynostosis and potential mesenchymal stem cell-based suture regeneration therapeutic strategies.

## Introduction

**Fig. 1.**
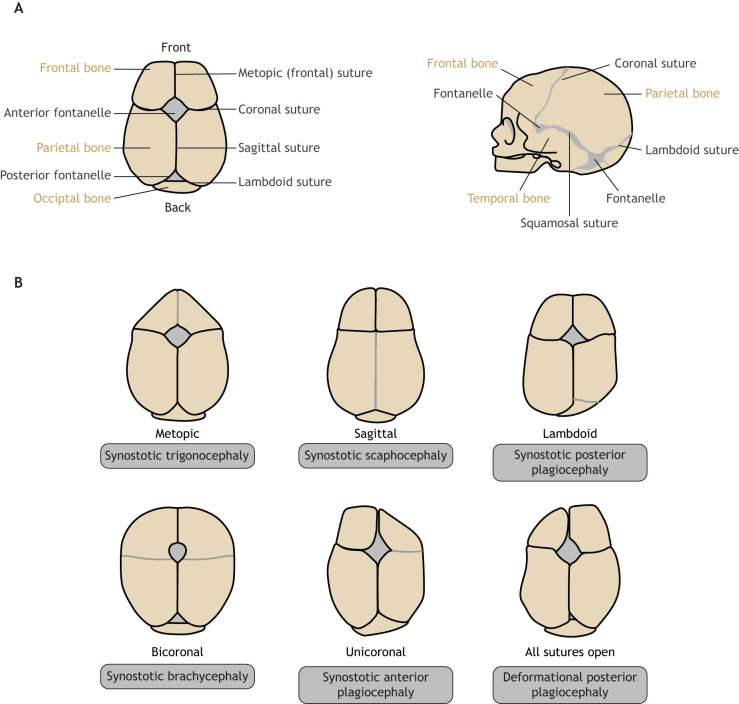
**Cranial sutures and craniosynostosis in humans.** (A) A normal human infant skull shown from above (left) and human infant skull shown from the side (right). (B) Skull deformities caused by different forms of craniosynostosis. See Glossary (Box 1) for description of medical terms. Figure adapted from Buchanan et al. (2017) under the terms of the CC BY-NC 3.0 license.

Cranial sutures of the skull vault – including the metopic, coronal, lambdoid and sagittal sutures – are fibrous joints that connect the skull bones, coordinate growth and development of the skull and the brain, enable minor movements and serve as shock absorbers. Most calvarial sutures lie at neural crest–mesoderm boundaries, with two exceptions: the metopic suture (see Glossary, Box 1), which lies between the two neural crest-derived frontal bones, and the lambdoid suture, which lies between the two mesoderm-derived parietal and occipital bones (Balczerski et al., 2012; Di Ieva et al., 2013; Jiang et al., 2002; Lenton et al., 2005; Morriss-Kay and Wilkie, 2005). The metopic suture typically fuses by 9 months of age in humans (Vu et al., 2001). Other sutures fuse in adulthood, from the sagittal suture at ∼22 years of age to the squamosal suture at ∼60 years (Idriz et al., 2015). As the brain grows, the patent (i.e. non-ossified) calvarial sutures allow the skull to grow simultaneously (Fig. 1A). This process is essential for proper brain development (Morriss-Kay and Wilkie, 2005), as it accommodates postnatal brain enlargement (Nieman et al., 2012; Sharma, 2013).

**Box 1. Glossary**

**Acrocephaly:** a congenital abnormality in which the top of the skull presents with a conical shape.

**Amblyopia:** colloquially known as ‘lazy eye’ and characterized by poor vision in one eye as a result of abnormal vision development.

**Biparietal diameter:** the maximum diameter of the fetal skull at the eminences of the parietal bones; this is one of the basic parameters used to assess fetal skull size.

**Brachycephaly:** a shortened skull shape with a shorter than normal length-to-width ratio.

**Crouzonoid facies:** common features present in patients with Crouzon syndrome, including midface hypoplasia, a convex nose, a prognathic mandible, exophthalmos and posteriorly angled ears.

**Deformational plagiocephaly:** when an infant develops a flattened posterior of the skull or spot on the side of the head due to prolonged positioning/pressure on that area.

**Gain-of-function mutation:** a mutation that leads to increased or new activity of a gene product.

**Haploinsufficiency:** when one copy of a gene is inactivated or deleted, and the remaining copy is unable to appropriately compensate to maintain the expression levels required for normal function.

**Hypomorphic mutation:** a mutation that confers a gene product with a decreased activity level.

**Loss-of-function mutation:** a mutation that leads to decreased or ablated activity of a gene product.

**Metopic suture:** the fibrous joint that connects the two frontal bones.

**Otitis media with effusion:** a collection of fluid in the middle ear in absence of an ear infection.

**Papilledema:** swelling of the optic disc due to increased intracranial pressure.

**Plagiocephaly:** flattening of the posterior skull caused by lambdoid synostosis.

**Scaphocephaly:** long and narrow ‘boat-shaped’ head caused by sagittal suture fusion.

**Trigonocephaly:** triangularly shaped forehead caused by metopic suture fusion.

**X-linked mutation:** a mutation that occurs on the X chromosome; for this reason, males are more likely to be affected by these mutations as they only carry one X chromosome.

**Box 2. Craniosynostosis-associated disorders. (See Table 1 for genetic abnormalities and modes of inheritance associated with each disorder.)**

**Apert syndrome:** a rare genetic condition characterized by craniosynostosis and distinctive malformations of the skull, hands, feet and face. Affected children often also have intellectual disability.

**Baller-Gerold syndrome:** a rare condition characterized by craniosynostosis (most commonly coronal), bulging eyes, a prominent forehead and bone abnormalities.

**Beare–Stevenson syndrome:** a genetic disorder characterized by craniosynostosis, furrowed and wrinkled skin (cutis gyrate), bulging eyes and an underdeveloped maxilla.

**Bent bone dysplasia:** an often-lethal skeletal disorder characterized by craniosynostosis, osteopenia, hypoplastic pubic bone and clavicles, bent long bones and poor skull mineralization.

**Craniofrontonasal syndrome:** a rare condition characterized by coronal craniosynostosis, wide-set eyes, a cleft nose tip, a wide nasal bridge, and often cleft lip and/or palate.

**Crouzon syndrome:** a rare genetic disorder characterized by craniosynostosis, bulging eyes, a prominent forehead, midface hypoplasia and a short upper lip.

**Muenke syndrome:** a rare genetic disorder characterized by craniosynostosis, wide-set eyes and flattened cheekbones.

**Pfeiffer syndrome:** a rare genetic disorder characterized by craniosynostosis, and broad and medially deviated thumbs and big toes.

**Saethre-Chotzen syndrome:** a rare genetic condition characterized by coronal craniosynostosis, ptosis, a broad nasal bridge and widely spaced eyes. Affected children can also have small and rounded ears.

**Table 1.**
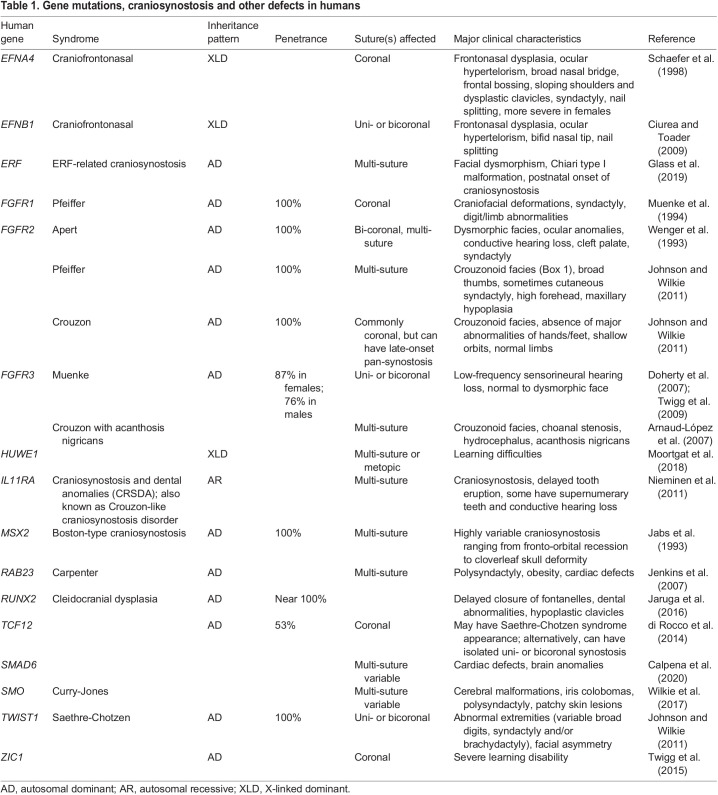
Gene mutations, craniosynostosis and other defects in humans

The premature fusion of cranial suture(s) leads to craniosynostosis, a major congenital craniofacial disorder that affects between 1 in 2100 and 1 in 2500 live births in the United States. Craniosynostosis can occur either as part of a recognized genetic syndrome or as an isolated pathology (Sharma, 2013). Infants with syndromic craniosynostosis may present with other deformities or symptoms, including neurological and respiratory problems (Johnson and Wilkie, 2011). The four main sutures implicated in craniosynostosis are the sagittal, coronal, metopic and lambdoid sutures. Defects in each of these sutures present with distinct clinical manifestations (Fig. 1B) that often require surgical repair.

Currently, craniosynostosis treatment is almost entirely surgical, sometimes paired with postoperative helmet therapy for maintenance (Wolfswinkel et al., 2021) (see Box 3). Corrective procedures are complex, long and associated with the risk of numerous complications, including heavy blood loss and its sequelae (Erb and Meier, 2016; Wolfswinkel et al., 2017). In some practices, 94% of patients are reported to be admitted to the intensive care unit after surgery (Wolfswinkel et al., 2017). Peri- and postoperatively, patients with syndromic craniosynostosis are significantly more likely to experience complications and re-synostosis than non-syndromic patients (Lee et al., 2012). Although surgery may restore normal appearance, in some cases, patients experience persistent deficits in intellectual ability and cognitive function, the severity of which partially depends on the type and extent of suture fused (Maliepaard et al., 2014; Speltz et al., 2015; Zechi-Ceide et al., 2012). Overall, there is a strong unmet medical need for effective therapies to treat craniosynostosis and to prevent re-synostosis.

**Box 3. Surgical treatment of craniosynostosis**

• The current standard treatment for craniosynostosis is surgery.

• Various approaches include distraction osteogenesis springs, endoscopically assisted strip craniectomy and open calvarial vault remodeling.

• Postoperative helmet therapy to help with skull reshaping can be implemented.

• More recently, technological advancements including machine learning, intraoperative near-infrared spectroscopy and 3D imaging have improved the accuracy of diagnostics and surgical planning.

• Surgical complications are common and include postoperative bleeding, puncture/lacerations, pneumonia, seizures, cardiorespiratory shock, hypovolemic shock, cerebrospinal fluid fistula and hematoma.

• Sutures can also re-fuse postoperatively, requiring surgical revision.

• The appropriate timing of surgical intervention is not well established, is currently case specific, and involves weighing the benefits and risks of early versus later intervention.

• Early intervention has been shown to improve neurological and developmental outcomes, yet it carries the risk of heavy blood loss and complications of general anesthesia, including possible neurodevelopmental defects.

• Many patients require reoperation driven by poor cosmetic outcomes, the desire for improved cranial morphology, re-synostosis and/or persistent increased intracranial pressure.

This Review aims to summarize our current understanding of the clinical types and manifestations of craniosynostosis in humans and provide an overview of calvarial suture development, as well as genetic and environmental risk factors implicated in the etiologies of craniosynostosis. We discuss the importance of animal models in pathophysiological studies of suture development and craniosynostosis. We also highlight the complexity and shortcomings of current surgical treatment and the ongoing efforts to develop less invasive and/or biological strategies in animal models. We review and critique recent studies that have identified various populations of suture mesenchymal stem cells (MSCs) that could improve the treatment of craniosynostosis. This knowledge will allow the field to better understand the pathogenesis of craniosynostosis and to develop improved mechanism-based biological therapies to treat patients.

## The types and clinical manifestations of craniosynostosis

### Craniosynostosis types and cranial phenotypes

Craniosynostosis is seldom diagnosed prenatally; it is most often identified through physical examination in the months after birth. However, some head shape and growth patterns can suggest syndromic craniosynostosis prenatally (Johnson and Wilkie, 2011). For example, severe forms of Pfeiffer syndrome (Box 2, Table 1) can commonly be identified via ultrasonography due to the characteristic cloverleaf skull appearance. Apert syndrome (Box 2) can also be indicated prenatally based on biparietal diameter (Box 1) and the presence of acrocephaly (Box 1) (Harada et al., 2019).

Craniosynostosis types and their corresponding phenotypes are depicted in Fig. 1B. The most common form of craniosynostosis is fusion of the sagittal suture, which is responsible for 45% of cases in the United States (Kolar, 2011). The primary outcome of sagittal suture fusion is an elongated head shape (scaphocephaly; Box 1) due to compensatory anterior–posterior lengthening (Dempsey et al., 2019). In ∼20-25% of patients with craniosynostosis, the coronal suture is affected, typically on only one side, and is called unilateral or unicoronal synostosis (Cohen, 2000). Unicoronal synostosis is characterized by ipsilateral forehead flattening and a rotation (‘twist’ or ‘yaw’) of the midface. In bilateral coronal synostosis, anterior–posterior growth is restricted with compensatory temporal expansion, leading to brachycephaly (Box 1) (Dempsey et al., 2019). Most bicoronal cases can be attributed to a known genetic cause (Vinchon, 2019). The next most common form is metopic craniosynostosis, which is responsible for ∼5-15% of cases (Cohen, 2000) and which leads to trigonocephaly (Box 1; a triangular-shaped forehead) and to narrowing of the temporal region (Dempsey et al., 2019). Finally, lambdoid synostosis accounts for less than 5% of cases (Cohen, 2000; Hentz and Mathes, 2006). Unilateral lambdoid suture fusion typically presents with bulging of the mastoid process and lowering of the cranial base, as well as displacement of the ear inferiorly and posteriorly (Dempsey et al., 2019). Bilateral lambdoid craniosynostosis occurs less commonly and leads to a flat and widened occipital region with displacement of the ear inferiorly and anteriorly (Delashaw et al., 1991). Plagiocephaly (Box 1), which is seen in lambdoid synostosis, should be distinguished from deformational plagiocephaly (Box 1). Deformational plagiocephaly is characterized by a flattened posterior skull due to positional molding and is a much more common cause of occipital asymmetry than lambdoid craniosynostosis (Rhodes et al., 2014).

### Additional manifestations associated with craniosynostosis

As mentioned above, craniosynostosis can occur either as an isolated non-syndromic disorder or as part of a recognized genetic syndrome (Sharma, 2013). Infants with craniosynostosis may present with non-suture deformities or symptoms (Table 1, Box 2) (Johnson and Wilkie, 2011), such as hydrocephalus, hearing deficits, visual impairment, increased intracranial pressure (ICP) or neurocognitive dysfunction-related intellectual disabilities (IDs). Hydrocephalus occurs in 12-15% of syndromic craniosynostosis patients (Cinalli et al., 1998; Di Rocco et al., 2011). This can be due to obstructed cerebrospinal fluid (CSF) outflow from an abnormally small posterior skull with subsequent constriction of the fourth ventricle or to impaired CSF absorption (Collmann et al., 2005). Hearing deficits are frequently associated with craniosynostosis (Agochukwu et al., 2014; Biamino et al., 2016) and manifest as conductive hearing loss and otitis media with effusion (Box 1). Both result from the narrowing and abnormal angulation of the Eustachian tube due to the narrowing and contour of the skull base (Couloigner and Ayari Khalfallah, 2019). Visual pathologies are more common in syndromic (27%) relative to non-syndromic (17%) patients (Tunçbilek et al., 2010). The most common causes of vision loss in syndromic craniosynostosis are amblyopia (Box 1) and optic neuropathy; the latter results from increased ICP, which causes papilledema (Box 1) (Tay et al., 2006; Touzé et al., 2019). In general, these abnormalities are more prevalent or severe in syndromic relative to non-syndromic craniosynostosis.

Patients with syndromic craniosynostosis are significantly more likely to have ID, social problems and attention deficits, and to show inhibited and withdrawn behaviors. Such neurocognitive deficits typically become apparent once a child begins schooling, at ∼6-7 years of age (Da Costa et al., 2006; Maliepaard et al., 2014). Although the prevalence and severity of ID vary across syndromes and patients, Apert syndrome has the highest rate of ID (Da Costa et al., 2006; Lajeunie et al., 1999; Lefebvre et al., 1986; Patton et al., 1988; Renier et al., 1996, 2000; Sarimski, 1997). Non-syndromic patients are significantly less likely to present with ID (Da Costa et al., 2006). The majority of studies have demonstrated that, in patients with non-syndromic craniosynostosis, intelligence typically remains within the normal range long term (Arnaud et al., 1995; Magge et al., 2002; Shipster et al., 2003), although some patients may have ID (Sidoti et al., 1996; Speltz et al., 2015).

The premature fusion of cranial sutures restricts brain growth, leading to abnormal brain structure (i.e. microcephaly) and to abnormal cognitive functions in craniosynostosis. However, neurocognitive deficits have been largely neglected in basic research on craniosynostosis (Esparza and Hinojosa, 2008; Grova et al., 2012; Lee et al., 2012, 2019). The etiologies of the anatomical and neurocognitive abnormalities seen in craniosynostosis patients largely remain unknown, raising questions as to how current clinical treatments and their timing can improve patients' neurological deficits. For example, the roles that different therapeutic interventions might play in attenuating the later-observed cognitive deficits, including behavioral, linguistic or visuospatial difficulties, are unclear (Brooks et al., 2018; Esparza and Hinojosa, 2008).

Increased ICP on the cerebral cortex is thought to contribute to impaired neurocognition and intelligence (Morriss-Kay and Wilkie, 2005). This is supported by the fact that both syndromic and non-syndromic craniosynostosis patients display increased ICP (Erb and Meier, 2016; Tamburrini et al., 2005; Wolfswinkel et al., 2021) and that higher ICP statistically correlates with poorer performance on psychometric testing (Renier et al., 1982; Wolfswinkel et al., 2021). Given the crucial roles of ICP in impaired neurocognitive functions, as revealed by mouse models of craniosynostosis and as discussed later in this Review, it is important to know the cause of elevated ICP in craniosynostosis. Historically, elevated ICP is mainly assumed to result from the premature fusion of the skull, reducing the volume that the brain can occupy and leading to its compression (Johnson and Wilkie, 2011).

However, patients continue to have chronic elevated ICP even after surgical skull expansion, which suggests that there are other causes of ICP elevation in craniosynostosis. Accumulating evidence suggests that ICP elevation might partly result from venous malformations that lead to CSF accumulation and to elevated ICP (Cinalli et al., 1998; Di Rocco et al., 2011; Harada et al., 2019; Hayward, 2005; Stevens et al., 2007; Taylor et al., 2001). For example, in patients with complex craniosynostosis, venous malformations that particularly affect the sigmoid–jugular region appear to be the main contributors to increased ICP (Taylor et al., 2001). Such abnormalities and subsequent venous hypertension can, in turn, impair CSF absorption and flow (Hayward, 2005). Further evidence that CSF absorption contributes to increased ICP, independent of calvarial vault size, is provided by clinical reports that increased ICP is more common among patients with midline (i.e. sagittal) rather than coronal synostosis (Thompson et al., 1995a,b). It is postulated that the unusually high ICP in sagittal synostosis patients may arise from this suture's unique ability to compress the sagittal sinus and/or interfere with the arachnoid granulations' drainage into the sinus (Choi et al., 2016).

Indeed, the roles and mechanisms of cerebral vein malformations in ICP and in neurocognitive dysfunction warrant further investigation. Additional open questions in craniosynostosis include whether diffuse malformation of the central nervous system, intrinsic brain dysfunction, brain metabolic defects and/or hemodynamic instability might also contribute to the learning disabilities and cognitive deficits of craniosynostosis patients, independently of the physical compression of the brain imposed by the prematurely fused skull (Brooks et al., 2018).

## Cranial suture development

Cranial suture development is a complex process that is coordinated with skull bone and brain development. A better understanding of the molecular and cellular regulatory mechanisms of cranial suture development is crucial for development of innovative treatments for craniosynostosis.

As calvarial bones develop, migratory mesenchymal cells contribute to suture formation. These mesenchymal cells originate from either head mesoderm or cranial neural crest cells. Furthermore, cranial sutures are often positioned at the boundary of cranial neural crest- and mesoderm-derived bony elements (Ishii, et al., 2015; White et al., 2021).

Studies have shown that newborns with Apert syndrome have cranial suture developmental defects at 15 weeks of gestation, not long after the coronal sutures first become apparent (Mathijssen et al., 1999). This suggests that developmental defects in craniosynostosis occur at the early stages of coronal suture development. In 2012, Deckelbaum et al. used a mouse model to demonstrate the presence of coronal suture precursors in head mesoderm at embryonic day (E)7.5-E8.0 (Deckelbaum et al., 2012). These precursors ultimately migrate to the supraorbital ridge between E8.5 and E9.0, followed by apical migration between E11.5 and E13.5. By E13.5, these cells give rise to the coronal suture and the overlapping parietal and frontal bones. The osteogenic fronts consist of fibroblast-like cells undergoing differentiation that are responsible for osteogenesis as the skull expands to accommodate the growing brain (Ishii et al., 2015). Overall, loss of boundary integrity between cranial neural crest- and mesoderm-derived cells, abnormal specification of suture cells during embryonic development and premature loss of mesenchymal stem cells in the suture at a later stage may constitute the cellular mechanisms of craniosynostosis (Ishii et al., 2015; White et al., 2021).

Regarding the molecular mechanisms of craniosynostosis, numerous mutations can contribute to dysregulation of suture mesenchymal sells and subsequent premature suture fusion. One such gene is twist family bHLH transcription factor 1 (*TWIST1*), which encodes a basic helix-loop-helix transcription factor. Heterozygous loss-of-function mutations (Box 1) in *TWIST1* lead to Saethre-Chotzen syndrome in humans (Box 2, Table 1). Cre-based lineage tracing studies in mice identified that *Twist1* haploinsufficiency (Box 1) leads to a loss of the osteogeneic–non-osteogenic boundary and subsequent ossification of the coronal suture, typically between postnatal day (P)9 and P13 (Behr et al., 2011; Merrill et al., 2006). *Twist1* mutant mice aberrantly express Notch2 in the suture mesenchyme; this early marker of osteogenesis is not normally expressed in this cell population (Ishii et al., 2015). Further, *Twist1* mutants have reduced ephrin A4 (*Efna4*) expression. Ephrin–Eph signaling has well-documented roles in boundary formation. Coupled with the finding that loss-of-function *EFNA4* mutations in humans lead to coronal synostosis, it is postulated to be a downstream effector of Twist1 in loss of boundary integrity (Merrill et al., 2006). Similarly, ephrin B1 (*EFNB1*), a gene implicated in a craniosynostosis disorder known as craniofrontonasal syndrome, is predicted to be important in boundary maintenance. Gain-of-function mutation (Box 1) in msh homeobox 2 (*MSX2*) causes Boston-type craniosynostosis. *Msx2* expression has been identified in the osteogenic front (Jabs et al., 1993), and is postulated to be involved in bone deposition and resorption.

**Fig. 2.**
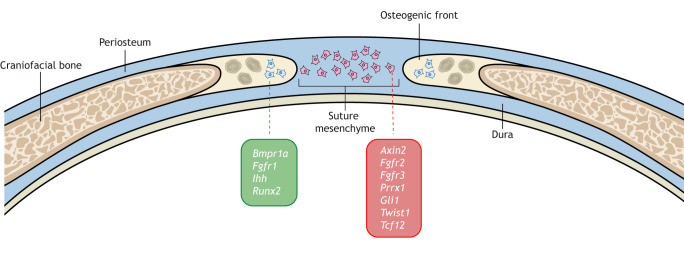
**Suture development at postnatal stage.** A cross-section schematic of the coronal suture that highlights some genes that are expressed in the suture mesenchyme versus the ones that are expressed at the osteogenic front. These gene expression patterns are dynamic and may change throughout postnatal suture development.

Gain-of-function mutation in RUNX family transcription factor 2 (*RUNX2*) is also responsible for craniosynostosis. RUNX2 is known to be inhibited by TWIST1 (Kronenberg, 2004) and is involved in the regulation of osteoblast differentiation (Komori, 2018), implicating it in the pathogenesis of premature suture fusion (Fig. 2). Reduced expression of ETS2 repressor factor (*ERF*) has also been found to cause multi-suture craniosynostosis. Studies in mice have identified the ability of Erf to inhibit Runx2 activity, and thus subsequent osteogenesis and suture homeostasis (Twigg et al., 2013).

Heterozygous mutation of transcription factor 12 (*TCF12*), like that of *TWIST1*, can lead to the development of Saethre-Chotzen syndrome (Sharma et al., 2013). Tcf12 forms a heterodimer with Twist1 (Connerney et al., 2006), and compound heterozygosity generates a more severe craniosynostosis phenotype (Sharma, 2013). Tcf12 has been found, like Twist1, to function in murine embryonic suture formation. In addition, loss of *Tcf12* contributes to decreased asymmetry of the mesenchymal cells in the parietal and frontal bones of the coronal suture, suggesting that it may have an important role in maintaining the overlap of these two bones and thus preventing synostosis (Ting et al., 2022). Together, these findings provide evidence for a crucial interaction between Twist1 and Tcf12 in regulating suture development.

In humans, loss-of-function mutation of RAB23, member RAS oncogene family (*RAB23*) leads to Carpenter syndrome with multi-suture synostosis, polysyndactyly, obesity and cardiac defects (Jenkins et al., 2007). It has been suggested that the role of RAB23 in the pathogenesis of craniosynostosis involves negative regulation of both FGFR and Hedgehog–GLI1 signaling, which are involved in osteoprogenitor cell recruitment and stem cell maintenance within the sutures (Hasan et al., 2020).

Missense mutations in interleukin-11 receptor alpha subunit (*IL11RA*) have been implicated in Crouzon-like craniosynostosis, which is characterized by multiple suture fusions and dental anomalies, such as delayed tooth eruption or supernumerary teeth. Interleukin 11 (IL-11) is a cytokine involved in bone remodeling that functions by activating cells via the IL-11 receptor (Agthe et al., 2018).

More recently, mutations in Zic family member 1 (*ZIC1*); SMAD family member 6 (*SMAD6*); HECT, UBA and WWE domain-containing 1, E3 ubiquitin protein ligase (*HUWE1*); and smoothened, frizzled class receptor (*SMO*) have been identified as causal in the development of craniosynostosis. However, their specific roles in suture development have not yet been investigated (Calpena et al., 2020; Moortgat et al., 2018; Twigg and Wilkie, 2015b; Wilkie et al., 2017).

Gain-of-function mutations in fibroblast growth factor receptor 1 (*FGFR1*), *FGFR2* and *FGFR3* are responsible for many craniosynostosis syndromes, including bent bone dysplasia and Crouzon, Apert, Pfieffer, Beare–Stevenson and Muenke syndromes, among others. The role of FGF/FGFR signaling is well established in controlling differentiation and proliferation of mesenchymal and ectodermal cells and in maintaining skeletal homeostasis (Moosa and Wollnik, 2016; Su et al., 2014).

**Fig. 3.**
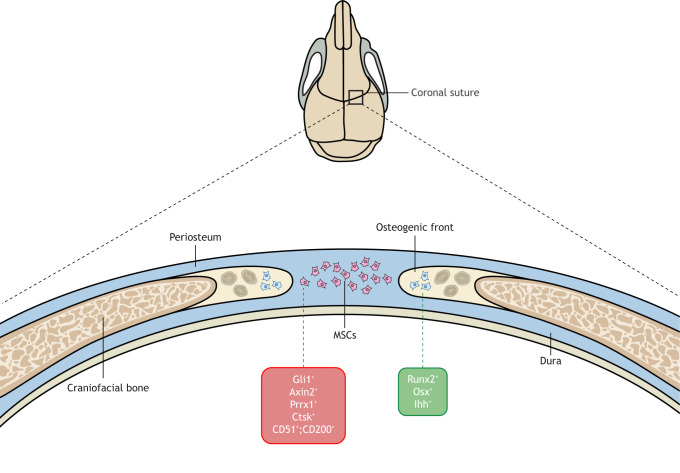
**Suture MSCs and their loss in animal models of craniosynostosis.** A cross-section schematic of the coronal suture of a mouse, showing cells in the suture mesenchyme and in the osteogenic front. Genetic lineage tracing and surface marker purification studies have identified multiple populations of suture mesenchymal stem cells (MSCs), including Gli1^+^, Axin2^+^, Prrx1^+^, Ctsk*^+^* and CD51^+^;CD200^+^ cells. These cells maintain suture patency and coordinate appropriate development. *Twist1^+/−^* craniosynostosis mouse embryos lose Gli1^+^ and CD51^+^;CD200^+^ suture MSCs during postnatal stages of development, and suture MSCs undergo premature differentiation into osteogenic cells, which results in premature suture synostosis.

The cranial suture harbors an MSC niche that is crucial for craniofacial homeostasis and repair (Zhao et al., 2015). Zhao et al. identified MSCs that express Gli1 in patent adult mouse sutures (Zhao et al., 2015). Lineage tracing showed that these cells give rise to the osteogenic front, dura, periosteum and calvarial bones under normal conditions and upon injury. Two groups independently used mouse models to show that, like Gli1^+^ cells, Axin2- and Prrx1-expressing cells act as postnatal suture MSCs and contribute to calvarial bone development and to regeneration (Maruyama et al., 2016; Wilk et al., 2017; Zhao et al., 2015). A separate study used the skeletal-targeting Cre driver, cathepsin K-Cre (*Ctsk^Cre^*), to label periosteal mesenchyme and identified a population of periosteal stem cells that are also present in the calvarium in mice; however, the functions of Ctsk*^+^* cells in the cranial suture have yet to be defined (Debnath et al., 2018). Although the above studies used lineage tracing to study suture MSCs *in vivo*, a recent study used a panel of surface markers (CD51^+^, CD200^+^, CD45^−^, Ter119^−^, Tie2^−^, Thy1.1^−^, Thy1.2^−^, 6C3^−^, CD105^−^) and identified a population of CD51^+^;CD200^+^ skeletal stem/progenitor cells in mouse cranial sutures (Menon et al., 2021) (Fig. 3).

*In vitro* and *in vivo* functional studies suggest that the different suture MSC lineages are heterogeneous, can self-renew and undergo tri-lineage differentiation into bone, cartilage and adipogenic stromal cells, and can participate in cranial suture development, homeostasis and bone regeneration (Holmes et al., 2021; Zhao et al., 2015). Fundamental questions remain in the field. For example, how do these different populations of suture MSCs, including Gli1^+^, Axin2^+^, Prrx1^+^, Ctsk*^+^* and CD51^+^;CD200^+^ cells, relate to each other? Do they act in a hierarchy of lineage-restricted stem cells, as in the hematopoietic system? Or do they localize in distinct spatial regions and act in parallel during suture development and homeostasis?

There are several new directions in the study of molecular and cellular regulatory mechanisms of cranial suture formation and tissue homeostasis under normal and disease conditions. First, how are sutures formed and how is their patency maintained? Do the aforementioned key markers affect the fate of MSCs in development and tissue homeostasis of cranial sutures and, if so, how? With cell lineage tracing and gene expression analysis at single-cell resolution, we are discovering a heterogeneous MSC population that contributes to the formation and maintenance of cranial sutures (Holmes et al., 2021; Li et al., 2021). Second, mechanical transduction of either tensile or compressive loading can significantly affect suture morphology (Herring, 2008). As brain growth exerts a force on the skull, how this force affects suture MSCs will be an important area of investigation. Third, it has been proposed that suture growth during brain development is regulated by various secreted factors (Twigg and Wilkie, 2015a). Dura-mediated signaling also influences suture development. The dynamic interaction between suture MSCs and the dura or periosteum will also be an important focus of our future study.

## Etiology of craniosynostosis

### Human genetics in craniosynostosis

The identification of pathogenic mutations in syndromic craniosynostosis patients has provided tremendous benefits for genetic consulting, diagnostic testing, risk assessment, reproductive advice, prognostic guidance, treatment planning, and disease mechanism studies. Overall, the genetic basis for craniosynostosis can be identified in only ∼20-30% of patients; 86% of these identifiable genetic causes consist of single-gene mutations and the remainder are chromosomal abnormalities (Johnson and Wilkie, 2011). The inheritance patterns of causative mutations are typically autosomal dominant. They are also less commonly X-linked (Box 1; e.g. craniofrontonasal syndrome) or recessive (e.g. Baller-Gerold syndrome) (Lajeunie et al., 2005) mutations (Box 2, Table 1). The mechanism of these mutations in humans is seldom a complete loss of function, as total functional loss is likely to be lethal *in utero*, given that the mutated gene might be crucial for organogenesis early in embryonic development. By comparison, mammalian skull growth and cranial suture formation occur relatively late in embryogenesis. Thus, the genetic mechanisms of craniosynostosis instead often involve haploinsufficiency, dominant gain-of-function or recessive hypomorphic mutations (Box 1), and are occasionally X linked (Twigg and Wilkie, 2015a). Table 1 shows the common genes implicated in craniosynostosis. Some mutations are associated with syndromic craniosynostosis (Table 1), while others contribute to non-syndromic suture-fusion phenotypes (Calpena et al., 2020; Cornille et al., 2019; Johnson and Wilkie, 2011; Twigg and Wilkie, 2015a; Wilkie et al., 2017). Craniosynostosis is associated with over 180 syndromes (Durham et al., 2017), and this list is certainly non-exhaustive.

Mutations associated with familial craniosynostosis, including those in FGF/FGFR pathway genes, *TWIST1* and *EFNB1*, among others, often occur in genes with critical functions in craniofacial and/or skeletal morphogenesis, and are thus highly penetrant or cause severe clinical abnormalities in patients. An alternative method to identify potential roles for common disease-modifying genes in craniosynostosis that might confer a relatively modest risk is the genome-wide association study (GWAS). The first GWAS on craniosynostosis identified susceptibility loci for non-syndromic sagittal craniosynostosis near bone morphogenetic protein-2 (*BMP2*) and within Bardet-Biedl syndrome 9 (*BBS9*) (Justice et al., 2012). The same group recently performed an additional GWAS, which implicated the *BMP7* locus as a risk factor for non-syndromic metopic craniosynostosis (Justice et al., 2020). Exome sequencing of parent–offspring trios identified *SMAD6* mutations with incomplete (<60%) penetrance causing non-syndromic midline (sagittal and metopic) craniosynostosis. Interestingly, all *SMAD6* mutations associated with aberrant phenotypes are associated with a *BMP2* mutant allele identified from previous GWAS (Timberlake et al., 2016). These findings suggest a two-locus inheritance mechanism for non-syndromic craniosynostosis that involves the inheritance of rare *SMAD6* and of more common *BMP2* alleles. We foresee that advanced human genetic and genomic approaches will uncover new variants that will advance our understanding of disease mechanisms and inform the development of new therapies.

## Environmental risk factors in craniosynostosis

Causative mutations are identified in a minority of craniosynostosis patients. The etiology of non-syndromic craniosynostosis in most infants is unknown and most cases occur in families with no history of the disease (Yilmaz et al., 2019). Overall, 70-80% of all craniosynostosis cases manifest with no described genetic cause, which indicates that new disease-causing mutations remain to be identified, as do potential environmental factors or gene–environment interactions (Durham et al., 2017; Hentz and Mathes, 2006; Johnson and Wilkie, 2011; Vinchon, 2019). The idea that environmental risk factors contribute to the etiology of craniosynostosis is also consistent with clinical variability, such as asymmetric suture fusions and variable phenotypic penetrance. Even in syndromic craniosynostosis with defined genetic mutations, there is substantial variation in clinical manifestations, suggesting the potential involvement of environmental risk factors. For example, patients that are haploinsufficient for *TWIST1* develop Saethre-Chotzen syndrome (Box 2, Table 1), but display substantial phenotypic heterogeneity (Di Rocco et al., 2011; Lajeunie et al., 1995). This heterogeneity is also seen in *Twist1^+/−^* mice, as discussed later in this Review. A number of epidemiological studies have linked craniosynostosis to various environmental factors (Browne et al., 2011; Carmichael et al., 2008; Durham et al., 2017, 2019a; Grewal et al., 2008; Lajeunie et al., 2001; Rasmussen et al., 2007; Reefhuis et al., 2015, 2011, 2003; Sanchez-Lara et al., 2010; Sergesketter et al., 2019). These include maternal risk factors, such as diabetes (Durham et al., 2017; Sergesketter et al., 2019), smoking (Carmichael et al., 2008; Durham et al., 2017; Grewal et al., 2008; Hackshaw et al., 2011), high caffeine consumption (Browne et al., 2011) and thyroid disease (Rasmussen et al., 2007). The risk of craniosynostosis also increases in pregnant women taking certain medications, such as selective serotonin reuptake inhibitors (Durham et al., 2019a; Reefhuis et al., 2015) and clomiphene citrate, a medication used to treat infertility (Ardalan et al., 2012; Reefhuis et al., 2011, 2003). Below we discuss some of these factors in more detail.

### Diabetes

Mothers of infants with craniosynostosis have a significantly higher rate of gestational diabetes than mothers of non-affected infants (Sergesketter et al., 2019). Studies have demonstrated that 11.6-13.5% of mothers of children with craniosynostosis were diagnosed with diabetes mellitus during pregnancy (Ardalan et al., 2012; Sergesketter et al., 2019). This rate of gestational diabetes is significantly higher than that of mothers who have children without craniosynostosis (2.9%). Further, a case-control study found diabetes to be a significant risk factor for having a craniosynostotic child (Ardalan et al., 2012).

#### Smoking

Maternal smoking appears to be the greatest risk [odds ratio (OR) 1.6] to the developing fetus after the first trimester, and the risk increases with the number of cigarettes smoked per day (Carmichael et al., 2008). A large retrospective study of 173,687 children born with malformations and 11.7 million controls found that craniosynostosis is 33% more likely to occur in children whose mothers smoked during pregnancy than in those whose mothers who did not [OR 1.33; 95% confidence interval (CI) 1.03-1.73] (Hackshaw et al., 2011). A case-control study found maternal smokers to be 1.7 times (95% CI 1.2-2.7) more likely to have a child with craniosynostosis. In mothers who smoked more than one pack per day during pregnancy, the likelihood of having a child with craniosynostosis rose to 3.5 times that of non-smokers (Alderman et al., 1994). These studies provide evidence that smoking in pregnancy may be a risk factor for craniosynostosis.

#### Caffeine

A study by Browne et al. using data from the National Birth Defects Prevention Study demonstrated that mothers who consume high amounts of caffeine (≥300 mg per day) have significantly elevated relative odds (OR 1.34) of having an infant with craniosynostosis (Browne et al., 2011). This study used data from 797 craniosynostosis cases and adjusted for covariates including maternal age, race/ethnicity, education, body mass index, residence, fertility medication and sickness during pregnancy. Thus far, this is the only study conducted on the relationship between caffeine and the development of craniosynostosis, so more research is needed to elucidate caffeine's impact on cranial suture patency.

#### Thyroid disease

For years, the relationship between thyroid disease and craniosynostosis had been primarily presented in small case studies (Cove and Johnston, 1985; Daneman and Howard, 1980; Johnsonbaugh et al., 1978; Nishihara et al., 2006; Penfold and Simpson, 1975). More recently, a study conducted by Rasmussen et al. using data from the National Birth Defects Prevention Study determined that mothers with thyroid disease had an OR of 2.47 (95% CI 1.46-4.18), indicating a heightened risk of giving birth to a child with craniosynostosis (Rasmussen et al., 2007). Associations with thyroid disease appeared to vary by suture. Sagittal (OR 3.32; 95% CI 1.29-8.52) and coronal (OR 2.11; 95% CI 0.99-4.48) suture synostosis had the highest ORs of single-suture synostoses. However, the association between maternal thyroid disease and craniosynostosis was strongest for multi-suture involvement (OR 8.73; 95% CI 3.54-21.57) (Rasmussen et al., 2007). The results of this study suggest a relationship between thyroid disease and craniosynostosis; however, more investigation is necessary to gain a better understanding of this potential association.

#### Clomiphene citrate

Clomiphene citrate, a fertility drug used in early pregnancy, has been associated with craniosynostosis (Ardalan et al., 2012; Reefhuis et al., 2011, 2003). One case-control study on risk factors associated with both syndromic and non-syndromic craniosynostosis identified clomiphene citrate as one of the strongest independent risk factors (OR 12.71; 95% CI 1.42-113.6) (Ardalan et al., 2012). A study using data from the National Birth Defects Prevention Study (1997-2005) found that use of clomiphene citrate increased the odds of craniosynostosis development by 1.9 times (95% CI 1.2-3.0) after adjusting for potential confounders (Reefhuis et al., 2011).

Recent studies have also begun to investigate the impact of environmental factors on craniosynostosis in animal models (Durham et al., 2017, 2019a,b). For example, although mice exposed to nicotine *in utero* do not develop craniosynostosis, *in vitro* murine coronal suture cell cultures treated with nicotine show increased cell proliferation and altered calvarial growth (Durham et al., 2019b). *In utero* exposure to the serotonin selective reuptake inhibitor citalopram increased the incidence of craniosynostosis in mice and reduced the number of Gli1^+^ MSCs in the sutures (Durham et al., 2019a). These Gli1^+^ cells are integral to suture maintenance and their loss precedes suture fusion in the *Twist1^+/−^* mouse model of craniosynostosis (Zhao et al., 2015). Exposure *in utero* to excess thyroxine, a thyroid hormone involved in numerous metabolic functions, failed to alter the severity or frequency of the craniosynostosis phenotype seen in *Twist1^+/−^* mice, although it slightly affected skull shape (Durham et al., 2017). These results underscore the difficulty of studying environmental factors and gene–environment interactions in craniosynostosis.

Despite these tentative associations and experimental studies, the data associating environmental risk factors with the development of craniosynostosis remain inconclusive (Durham et al., 2017). We know little about which environmental risk factor(s) contribute to the phenotypic onset and progression of craniosynostosis or to what extent, or about how they differentially intersect with distinct genetic backgrounds to affect specific cell types and pathways that contribute to craniosynostosis. The effects of environmental factors might also vary depending on genotype, rendering some genetic variants important only in the presence of specific environmental factors. Thus, future efforts should seek to improve our understanding of the etiology and mechanisms of craniosynostosis that result from interactions between genetics and exposure to teratogens and other environmental factors.

## Animal models and developmental mechanisms of craniosynostosis

### Animal models of craniosynostosis

Multiple animal models are used to explore the mechanisms that underlie the pathophysiology of craniosynostosis. In particular, genetically modified mouse models have provided considerable insights into the molecular, cellular and developmental mechanisms of craniosynostosis, and their skulls share developmental and anatomical similarities with those of humans. Moreover, researchers have also leveraged the rapid embryonic development, external growth, and large and transparent embryos of zebrafish, which enable the visualization of development into adulthood *in vivo* (Grova et al., 2012). However, the zebrafish’s interfrontal suture remains patent throughout life, unlike the corresponding metopic and interfrontal sutures of humans and mice, respectively (Quarto and Longaker, 2005). Owing to its larger skull size, the rabbit is used to test therapeutic surgical manipulations and strategies (Grova et al., 2012). However, it is less genetically tractable than the mouse or zebrafish. Overall, the mouse is currently the most accurate animal model of craniosynostosis pathophysiology and is therefore the focus of this Review.

**Fig. 4.**
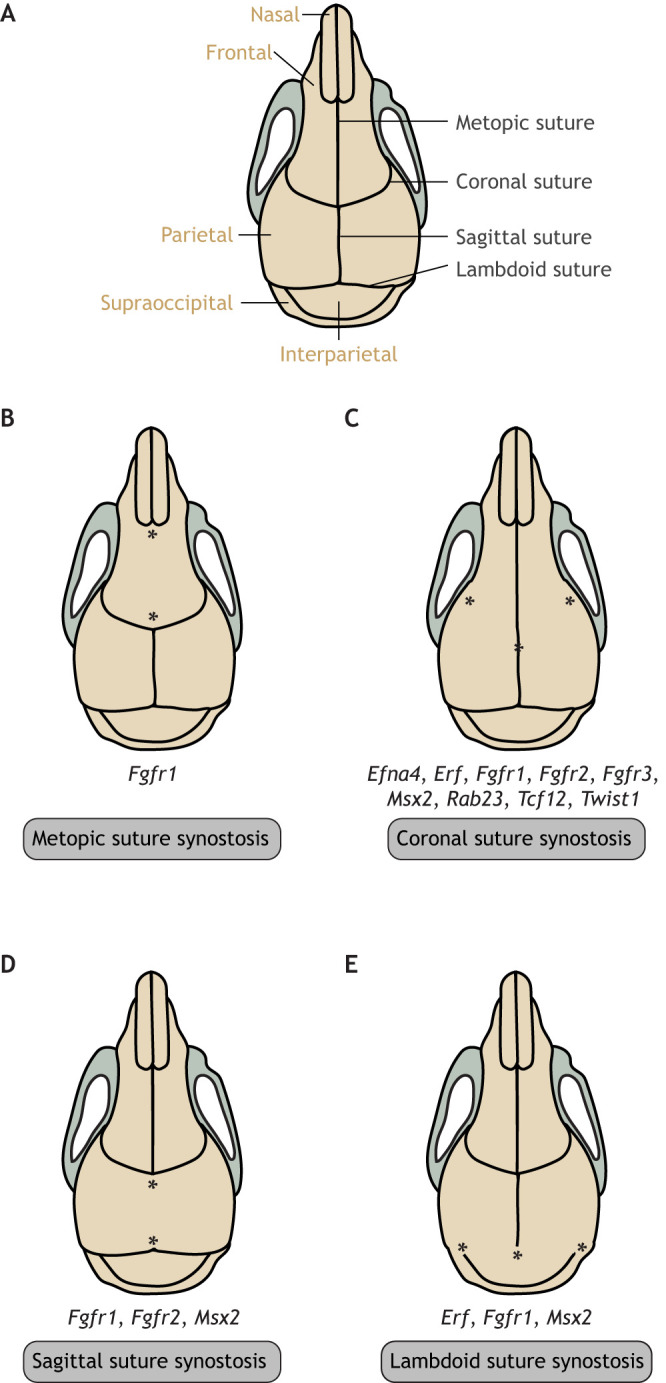
**The mutations associated with suture fusion in an adult mouse.** (A) Normal adult mouse skull. (B) Metopic (interfrontal) suture synostosis can be caused by *Fgfr1* mutation. (C) Coronal suture synostosis can be caused by mutation in *Efna4*, *Erf*, *Fgfr1*, *Fgfr2*, *Fgfr3*, *Msx2*, *Rab23*, *Tcf12* or *Twist1*. (D) Sagittal suture synostosis can be caused by mutation in *Fgfr1*, *Fgfr2* or *Msx2*. (E) Lambdoid suture synostosis can be caused by mutation in *Erf*, *Fgfr1* or *Msx2.* Asterisks indicate the corresponding fused sutures.

Craniosynostosis has been studied extensively in mouse models generated to mimic human disease variants (Fig. 4, Table 2). Some of our mechanistic insights into the etiology of craniosynostosis are derived from *Twist1^+/−^* and Fgf gain-of-function mouse models. For example, the same *TWIST1* haploinsufficiency in patients results in substantial phenotypic heterogeneity (Di Rocco et al., 2011; Lajeunie et al., 1995). *Twist1^+/−^* mice are a well-established model of craniosynostosis in Saethre-Chotzen syndrome and exhibit unilateral or bilateral coronal suture fusion (Fig. 4C) with incomplete penetrance (Table 2) (Behr et al., 2011; Bourgeois et al., 1998). This mouse model provides evidence for phenotypic heterogeneity, as only 40% of animals develop bilateral coronal suture fusion. Recent studies have shown that *Twist1^+/−^* mice with craniosynostosis also have elevated ICP and multiple neurocognitive behavioral abnormalities, including deficits in sociability, social memory, novel object recognition and motor learning (Yu et al., 2021). These studies highlight that craniosynostosis is a complex congenital disorder of multiple tissues/organs. Future studies could determine to what extent genetically modified mice could model non-suture defects identified in craniosynostosis patients.

**Table 2.**
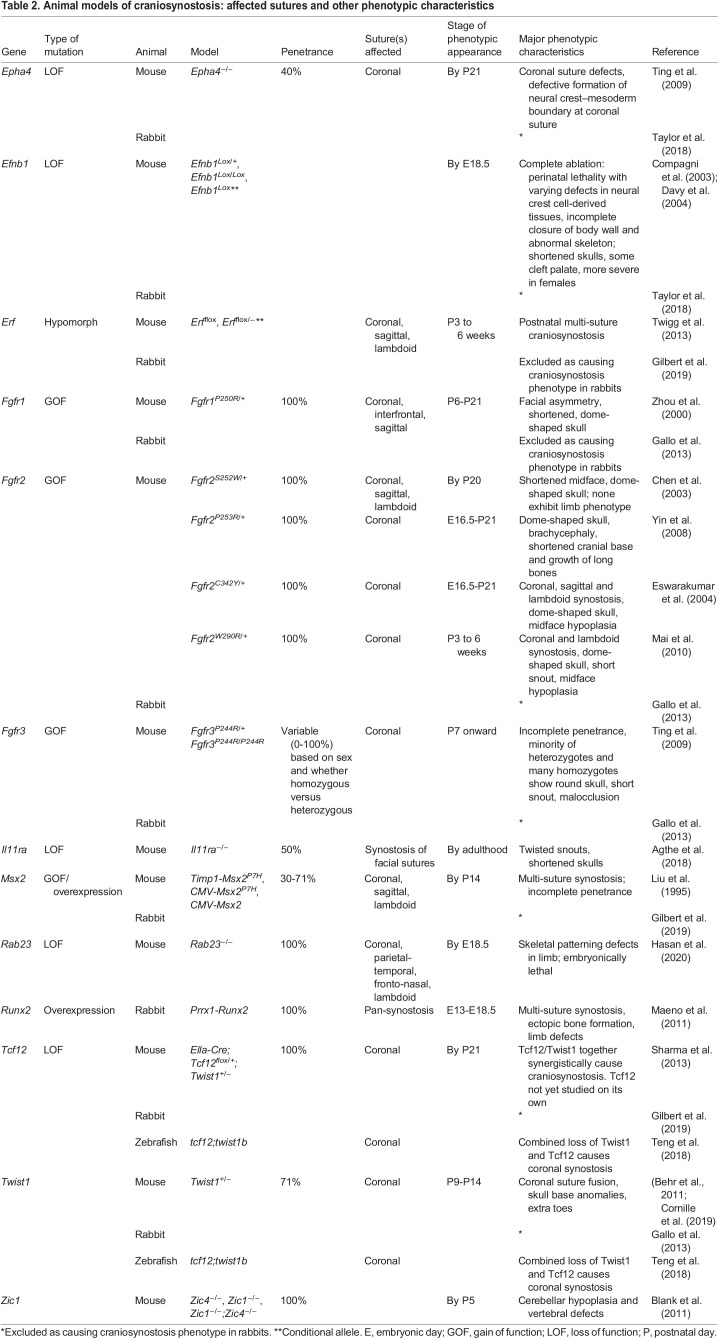
Animal models of craniosynostosis: affected sutures and other phenotypic characteristics

The contribution of gain-of-function mutations in the FGF signaling pathway to syndromic craniosynostosis has been well documented. The variety of phenotypes is contingent upon the tissue affected as well as the specific mutation. *FGFR2* gain-of-function mutations contribute to Apert syndrome, Beare–Stevenson syndrome, Crouzon syndrome, Pfeiffer syndrome and bent bone dysplasia (Box 2), each involving suture fusion (Jabs et al., 1994; Lajeunie et al., 1995; Merrill et al., 2012; Przylepa et al., 1996; Reardon et al., 1994; Rutland et al., 1995; Wilkie et al., 1995). Mutant mice carrying specific gain-of-function mutations in *Fgfr2* provide models of Apert syndrome. These mice have been engineered to carry *Fgfr2^S252W/+^* (Ser252Trp) (Chen et al., 2003) or *Fgfr2^253R/+^* (Pro253Arg) (Yin et al., 2008), which together constitute homologs of 97% of Apert syndrome cases in humans. In mice, these mutations result in premature fusion of the coronal suture, which is a key clinical feature of Apert syndrome (Park et al., 1995b). Mice carrying two additional gain-of-function *Fgfr2* mutations that can be found in humans with Crouzon syndrome – *Fgfr2^C342Y/+^* (Cys342Tyr) (Eswarakumar et al., 2004) and *Fgfr2^W290R/+^* – have phenotypes that recapitulate the clinical characteristics of this syndrome (Park et al., 1995a). *FGFR1* gain of function is primarily associated with Pfeiffer syndrome, which involves multi-suture synostosis (Muenke et al., 1994; Zhou et al., 2000). Mice carrying the P250R gain-of-function mutation in *Fgfr1* exhibit bicoronal synostosis, thus recapitulating the clinical features of Pfeiffer syndrome, as well as a dome-shaped skull and facial asymmetry (Cornille et al., 2019; Zhou et al., 2000). *FGFR3* gain-of-function mutation is responsible for both Muenke and Crouzon syndrome, and is associated with multi-suture synostosis (Wilkie et al., 2017) (Box 2, Table 1). Murine *Fgfr3^P244R/P244R^* (Pro244Arg) and *Fgfr3^P244R/+^* gain-of-function mutations have been generated to model Muenke syndrome and display variable phenotypes, with elevated penetrance of craniofacial abnormalities in homozygotes (Twigg et al., 2009). However, in comparison to previously described *Fgfr1* and *Fgfr2* mouse models of craniosynostosis, this one falls short of recapitulating the human disease; it has a very low craniofacial phenotypic penetrance with a sex bias opposite to what is seen in humans (Cornille et al., 2019). In addition to suture and skull defects, other disease symptoms can be modeled in these mice. For example, patients with Apert syndrome have craniosynostosis of varying sutures, syndactyly, ocular anomalies, conductive hearing loss, cleft palate and dental anomalies (Wenger et al., 1993). It is important to investigate how these mouse models can recapitulate this wide spectrum of abnormalities in Apert syndrome patients. Most of these additional clinical features seen in various craniosynostosis syndromes have yet to be extensively investigated. An exception to this is Muenke syndrome, for which studies have been conducted in mice to evaluate the ability of the *Fgfr3^P244R/+^* gain of function to produce sensorineural hearing loss like that seen in the human condition (Mansour et al., 2009).

A key limitation of these animal models is that a mutation in humans might result in different phenotypes in animal models, making it difficult to extrapolate the findings or investigate the mutation further. For example, some mouse models of disease-causing mutations, such as those causing Muenke syndrome, produce no phenotype or only a mild one (Holmes, 2012; Twigg and Wilkie, 2015a). These alterations are likely due to the species differences between humans and mice. Future *in vitro/ex vivo* human models or humanized chimeric mouse models of craniosynostosis should help with the translation of findings from animal models to humans.

## Developmental mechanisms in craniosynostosis

A key strength of the mouse models of craniosynostosis is that they can be used to investigate early developmental abnormalities, even before any anatomical changes become evident. For example, in mouse models of *Twist1^+/−^* craniosynostosis, the earliest detectable defect is the aberrant expression of Notch2, which is a marker of osteogenesis, and it is abnormally expressed in non-osteogenic suture mesenchymal regions in *Twist1^+/−^* mouse embryos at E12.5 (Yen et al., 2010). This finding identified that the premature osteogenic differentiation of suture MSCs is a disease mechanism of craniosynostosis in *Twist1^+/−^* mice. Studies of *Runx2* mutants have further supported this premature osteogenic differentiation of the suture mesenchyme in craniosynostosis. Runx2 is essential for osteoblastic differentiation and subsequent bone formation, and its duplication in humans leads to a craniosynostotic phenotype (Maeno et al., 2011), which is recapitulated in *Runx2* overexpression mouse models. These studies demonstrate that enhanced osteogenic differentiation, which occurs in *Twist1^+/−^* suture MSCs, contributes to craniosynostosis. More importantly, double heterozygotes for *Twist1* and *Runx2* deletion have none of the skull abnormalities observed in single-mutant mice (Bialek et al., 2004), which supports the notion that *Twist1^+/−^* haploinsufficiency promotes Runx2 activity and leads to the premature osteogenic differentiation of suture MSCs, ultimately resulting in craniosynostosis.

The loss of the cellular boundary in suture development is another developmental mechanism that contributes to craniosynostosis discovered in *Twist1^+/−^* mouse models. In *Twist1^+/−^* mice with coronal synostosis, the frontal–parietal boundary is defective at E14.5 and neural crest cells invade the undifferentiated mesoderm-derived cells in the coronal suture. This neural crest cell invasion is coupled with an increase in Msx2 expression and reduction in Efna4 expression (Merrill et al., 2006). *EFNA4* loss-of-function mutations in humans lead to craniofrontonasal syndrome with coronal synostosis (Ciurea and Toader, 2009; Schaefer et al., 1998). *Epha4^−/−^* mutant mice exhibit defects in the coronal suture and neural crest–mesoderm boundary that phenocopy those in *Twist1^+/−^* mice (Ting et al., 2009). Compound *Twist1^+/−^;Epha4^+/−^* heterozygotes have a more severe craniosynostotic phenotype than single-mutant heterozygous mice, as well as a higher penetrance of craniosynostosis. Labeling osteogenic precursor cells with 1,1′-dioctadecyl-3,3,3′,3′-tetramethylindocarbocyanine perchlorate, or DiI, a lipophilic membrane stain that diffuses to stain the entire cell, shows that *Twist1* and *Epha4* are required for the exclusion of such cells from the coronal suture. These results support a model in which the loss of integrity of the osteogenic–non-osteogenic boundary leads to osteogenic cells crossing into the suture regions, resulting in suture MSCs taking on an osteogenic fate.

In addition to embryonic development deficits, postnatal suture MSCs have been recently identified for their role in calvarial injury repair. Further, emerging evidence suggests that loss of suture MSCs could also be a disease-driving mechanism for craniosynostosis. It has been reported that Gli1^+^ cells were significantly reduced in the *Twist1^+/−^* craniosynostosis mouse model before the manifestation of suture closure defects; the ablation of this Gli1^+^ population resulted in craniosynostosis, indicating that the loss of Gli1^+^ cells is sufficient to cause craniosynostosis (Zhao et al., 2015). The population of CD51^+^;CD200^+^ cells is also significantly reduced in *Twist1^+/−^* mice before suture fusion defects occur (Menon et al., 2021), although it remains unknown whether ablation of this cell population leads to craniosynostosis in mice. It also remains unknown whether Axin2^+^ or Prrx1^+^ cells are reduced in craniosynostosis mouse models. In contrast to the ablation of Gli1^+^ cells, that of Prrx1^+^ cells does not result in a craniosynostosis phenotype (Wilk et al., 2017), indicating that Prrx1^+^ cells of the suture are dispensable for postnatal calvarial development. It is important to investigate how these suture MSCs behave and change their fates during the onset and progression of craniosynostosis. In addition, whether the lineage markers are mutually exclusive or whether cells can express more than one marker remains an open question. Such studies will provide insights into the regulation and functions of distinct suture MSCs under normal and disease conditions and will help guide stem cell-based therapeutic efforts to treat craniosynostosis and other craniofacial disorders.

## Prospective treatments for craniosynostosis

### Stem cell-based treatment

Given the shortcomings and complications of surgical intervention, as described above and in Box 3, preclinical studies have been conducted to develop alternative methods to address this unmet clinical need. One early study used autologous MSCs and the controlled release of transforming growth factor beta 3 (TGFβ3) during the operative correction of craniosynostosis (osteotomy) in rats (Moioli et al., 2008). This procedure successfully mitigated ossification and secondary synostosis following suture regeneration (Moioli et al., 2008). However, the independent replication of these findings has not been reported, and subsequent progress has had a limited impact on current clinical practice in treating craniosynostosis.

**Fig. 5.**
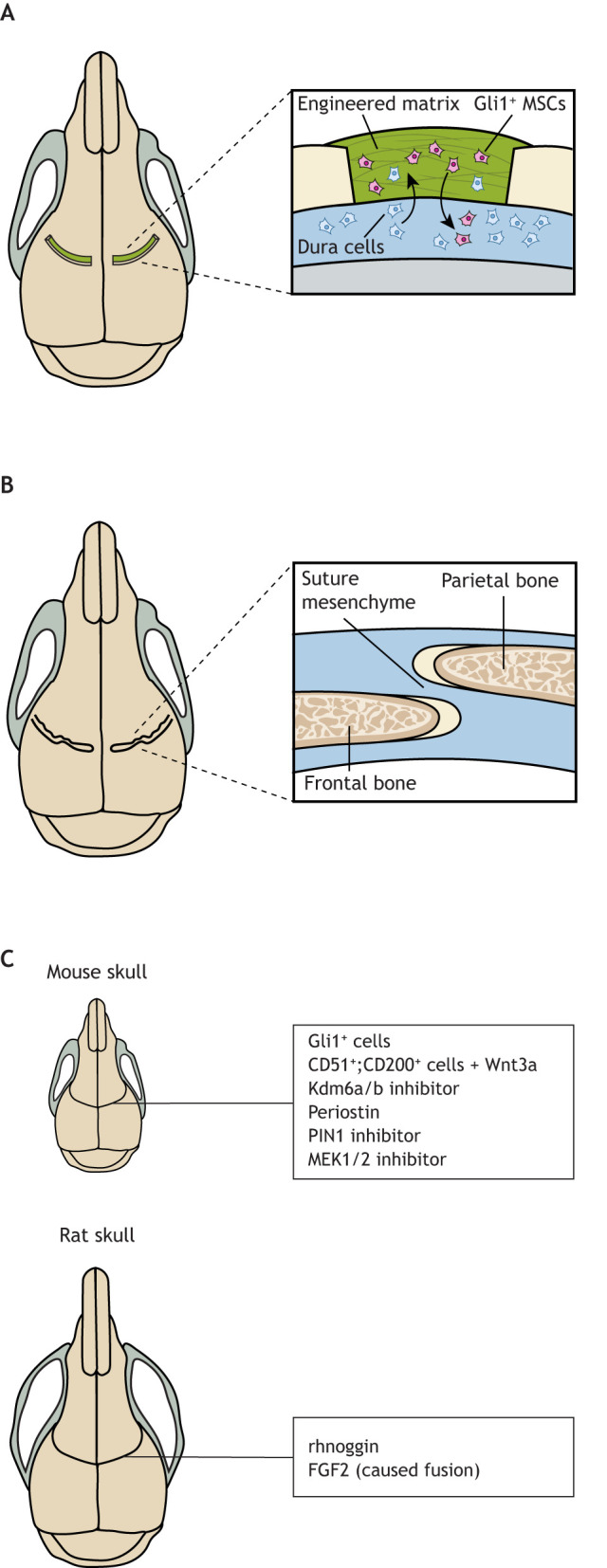
**Gli1^+^ MSC-mediated suture regeneration mitigates skull defects in craniosynostosis.** (A) A rectangular 0.3-0.4 mm-wide incision was made over each of the fused coronal sutures in *Twist1^+/−^* mice. *Ex vivo* Gli1^+^ MSCs were mixed with an engineered matrix composed of methacrylated gelatin, Matrigel and collagen I. This cell–matrix mixture was implanted into the incision. (B) The cell–matrix implant rescued skull deformity and, additionally, ameliorated defective brain structure and function in *Twist1^+/−^* mice with craniosynostosis. Moreover, this intervention regenerated normal suture morphology, and the regenerated sutures recapitulated those of wild-type mice in terms of their gene expression patterns and functions. Please see Yu et al. (2021) for more detail. (C) Various therapeutic approaches have been tested in mouse and rat models and have been shown to regenerate cranial sutures or prevent suture fusion. Specifically, Gli1^+^ MSCs or CD51^+^;CD200^+^ stem cells plus Wnt3a can support cranial suture regeneration in *Twist1^+/−^* mice. The Kdm6a/b inhibitor GSK-J4, recombinant periostin, the PIN1 inhibitor juglone, and the MEK1/2 inhibitor U0126 can prevent cranial suture fusion in mice. In the rat model, recombinant human (rh)noggin can prevent coronal suture fusion, whereas increased FGF2 activity can lead to (premature) suture fusion.

The first animal model of neurological dysfunctions in craniosynostosis was recently established using *Twist1^+/−^* mice (Jiang et al., 2002; Yu et al., 2021), a widely accepted model for Saethre-Chotzen syndromic craniosynostosis (Lee et al., 2012; Maliepaard et al., 2014; Speltz et al., 2015). This study added significant clinical value to this animal model as it provided a more comprehensive understanding of adverse effects caused by craniosynostosis, which has promising potential for guiding the development of new therapies that could ameliorate these deficits in patients. Importantly, our own group has recently implanted suture MSCs on resorbable scaffolds and succeeded in regenerating a functional suture while mitigating skull dysmorphology and neurological defects in mice (Yu et al., 2021) (Fig. 5). Specifically, removal of the fused suture followed by implantation of Gli1^+^ MSCs on a biodegradable scaffold could restore suture patency in *Twist1^+/−^* craniosynostosis mice. Importantly, this intervention normalized skull shape, neurocognitive function and the ICP of *Twist1^+/−^* mice. Such studies have been enabled by our previous identification of Gli1^+^ suture stem cells in the suture mesenchyme (Zhao et al., 2015), and by the finding that the premature loss of these cells might cause the premature fusion of the coronal suture in *Twist1^+/−^* mice.

This stem cell-based treatment of craniosynostosis has been further supported by a recent study in *Twist1^+/−^* mice, in which CD51^+^;CD200^+^ suture MSCs that were transplanted and treated with Wnt3a following suturectomy prevented re-synostosis (Menon et al., 2021). It is important to determine how implantation of CD51^+^;CD200^+^ and other suture stem cells (i.e. Axin2^+^, Prrx1^+^, Ctsk*^+^* cells) can regenerate cranial sutures, restore the skull and correct other associated anomalies in craniosynostosis.

These results highlight a potential paradigm shift in the future treatment of craniosynostosis, away from extensive surgery that incurs significant blood loss, to a less invasive stem cell-based biological solution. Most importantly, strategies that regenerate functional cranial sutures and that do not simply reshape the skull vault enable continued skull growth in coordination with brain development. They also normalize ICP, and thus are likely to reverse neurocognitive behavioral defects and eliminate the need for re-operation (Yu et al., 2021).

#### Pharmacological treatment

The therapeutic potential of numerous bioactive molecules has been assessed in craniosynostosis animal models. In the *Twist1^+/−^* mouse model of craniosynostosis, Pribadi et al. determined that expression of *Kdm6a* and *Kdm6b*, which encode chromatin modulators that are known promoters of osteogenesis, is upregulated in *Twist1^+/−^* calvarial cells (Pribadi et al., 2020). Using this information, they applied GSK-J4, a pharmacological inhibitor of *Kdm6a* and *Kdm6b*, which decreased osteogenic differentiation in *Twist1^+/−^* calvarial cells and reduced bone mineralization in *Twist1^+/−^* calvarial organ cultures. *In vivo*, GSK-J4 treatment prevented the fusion of coronal sutures in *Twist1^+/−^* mice.

Recombinant periostin, an extracellular matrix protein, is also reported to mitigate coronal craniosynostosis in *Twist1^+/−^* mice (Bai et al., 2018). Periostin is expressed in mesenchymal cells in the mouse skull and is repressed by Twist1. The results of this study demonstrated that periostin can reduce suture cell proliferation and differentiation through the upregulation of Wnt/β-catenin signaling. This mitigated coronal craniosynostosis in *Twist1^+/−^* mice.

Multiple studies have also targeted the role of FGFR in craniosynostosis. For example, intraperitoneal injection of the prolyl isomerase peptidyl-prolyl *cis-trans* isomerase interacting 1 (PIN1) inhibitor juglone into pregnant *Fgfr2^S252W/+^* mice from E14.5 to E18.5 prevents the development of Apert syndrome-like phenotypes in their offspring. PIN1 inhibition attenuates the elevated Runx2 levels seen in *Fgfr2^S252W/+^* mice, and Runx2 regulates Fgfr2 and Fgfr3 expression to induce the proliferation of osteoblast progenitor cells (Shin et al., 2018). Shukla et al. injected a MEK1/2 (also known as MAP2K1/2) inhibitor intraperitoneally into pregnant mice from E13.0 to E18.0 and found that this inhibition of MEK–ERK (also known as MAP2K–MAPK) signaling can also prevent *Fgfr2^S252W^*-related craniosynostosis and rescue skeletal abnormalities in the offspring of these mice (Shukla et al., 2007).

In rat models of craniosynostosis, recombinant human (rh)noggin protein was shown to inhibit coronal suture fusion (Shen et al., 2009). The decision to investigate noggin was based on its known expression in patent suture mesenchyme and its absence from the mesenchyme of fused cranial sutures. Increased FGF2 signaling, as seen in various craniosynostosis syndromes, suppresses noggin expression in the suture (Shen et al., 2009). Inappropriate expression of noggin is also implicated in the pathogenesis of FGFR-related craniosynostosis; implantation of human *FGFR2-*mutant osteoblasts treated with rhnoggin into chimeric nude rats prevented suture fusion, while implantation of *FGFR2-*mutant osteoblasts alone did not (Shen et al., 2009). This study provided evidence for noggin as an important mediator in the development of this disease and yet another possible future target for human therapies.

In another rat study, the authors modulated FGF activity *in utero* to assess its effects on the calvaria (Greenwald et al., 2001). The authors found that increased FGF2 activity resulted in the thickening of the parietal and frontal bones, as well as in coronal suture fusion. They postulated that stimulation of FGF activity led to significant changes of cellular proliferation, TGFβ1 expression and collagen I expression in the dura mater and suture; these molecular changes contributed to increased osteogenesis. Rabbit models have also been used to test bioactive molecules in craniosynostosis treatment, owing to their larger skull size. One study of rabbits with familial delayed-onset craniosynostosis found that treating 25-day-old animals with TGFβ3 combined with slow-absorbing collagen increased the width and area of the coronal suture (Chong et al., 2003). The exact mechanism behind these results has yet to be elucidated. However, there is evidence that isoforms of TGFβ mediate suture cell homeostasis (Opperman et al., 2002; Opperman and Ogle, 2002).

Another study performed suturectomies on rabbits at 10 days of age, then treated them with anti-TGFβ2 antibodies and found that the treated animals developed a significantly larger suture area than controls. Blocking the function of TGFβ2 not only kept sutures patent, but also improved craniofacial growth. Although the mechanism has not yet been fully investigated, the authors suggested, based on existing knowledge, that TGFβ2 was able to prevent postoperative re-synostosis by controlling the proliferation and apoptosis of cells surrounding the osteogenic fronts (Mooney et al., 2007) (Fig. 5).

Overall, most of these pharmacological studies focused on one time point of morphological change in cranial sutures. The long-term beneficial effects and pharmacokinetic profiles of these bioactive molecules have yet to be carefully evaluated, and their efficacy and biosafety in treating craniosynostosis remain obscure. But, in time, these studies might help to identify potential pharmacological targets for the future prevention and/or treatment of craniosynostosis.

## Conclusions and future directions

Despite research and technological advances improving clinical management of craniosynostosis, this congenital malformation remains challenging to manage and treat, both for patients and care providers. Given the complicated etiology of craniosynostosis, involving both genetic and environmental factors, more studies need to be conducted to elucidate this important relationship. Overall, the gene–environment crosstalk remains an understudied and important area, considering that only about a quarter of craniosynostosis cases currently have an identifiable genetic basis. Animal models carrying causative mutations coupled with exposure to potential environmental insults will be invaluable for us to gain a deeper understanding of the mechanisms of craniosynostosis.

Although craniosynostosis mainly affects the shape and size of the skull, it needs to be investigated in the context of brain development and function because sutures function as the sites of calvarial bone growth to accommodate the expansion of brain tissue in postnatal development. As discussed in this Review, deficits in brain structure and function are detected in a population of craniosynostosis patients. However, these brain-related defects have not been fully explored in many of the mutant animal models and will require further investigation. From an evolutionary perspective, cranial sutures may act as targets for change in craniofacial morphology and have contributed to the diversity of skull shapes across mammals and other vertebrates (White et al., 2021). As expansion of brain structure and function is key to human evolution, we need to expand our understanding of the connection between sutures, skull and brain function.

Another important area of future study is the cranial suture niche environment. Mesenchymal cells within each cranial suture contain functionally indispensable MSCs and are highly heterogeneous (Farmer et al., 2021; Holmes et al., 2021; Li et al., 2021; Zhao et al., 2015). Furthermore, adjacent structures such as the dura mater may contribute to the homeostasis of suture MSCs during development and in suture regeneration (Yu et al., 2021). Further studies are necessary to investigate the role(s) of other structures, such as blood vessels and nerves, in regulating tissue homeostasis within the suture.

Finally, how can regenerative medicine provide innovative treatment for patients with craniosynostosis? Suture MSC loss is a newly identified disease-driving mechanism. This discovery calls for a unique time window for therapeutic intervention. Proof-of-principle studies in mouse models show that replenishing Gli1^+^ MSCs within the suture niche after the defects have occurred can regenerate cranial suture and can mitigate both skull and neurocognitive defects. It will be informative to determine the feasibility and efficacy of this heterogenous suture MSC population in treating craniosynostosis, including Axin2^+^, Prrx1^+^, Ctsk^+^ and CD51^+^;CD200^+^ cells. Combining bioactive small molecule-based pharmacological approaches with suture MSC implantation is also a promising area for future research. Overall, pathophysiological studies will no doubt provide a better understanding of suture development, stem cell biology and tissue regeneration, and disease mechanism-based therapeutic efforts will help bring improved treatment for patients with craniosynostosis.
